# Runx Transcription Factors in T Cells—What Is Beyond Thymic Development?

**DOI:** 10.3389/fimmu.2021.701924

**Published:** 2021-08-06

**Authors:** Svetlana Korinfskaya, Sreeja Parameswaran, Matthew T. Weirauch, Artem Barski

**Affiliations:** ^1^Division of Allergy & Immunology, Cincinnati Children’s Hospital Medical Center, Cincinnati, OH, United States; ^2^Center for Autoimmune Genomics and Etiology, Cincinnati Children’s Hospital Medical Center, Cincinnati, OH, United States; ^3^Divisions of Biomedical Informatics and Developmental Biology, Cincinnati Children’s Hospital Medical Center, Cincinnati, OH, United States; ^4^Department of Pediatrics, University of Cincinnati College of Medicine, Cincinnati, OH, United States; ^5^Division of Human Genetics, Cincinnati Children’s Hospital Medical Center, Cincinnati, OH, United States

**Keywords:** RUNX1, RUNX2, RUNX3, cytokines, mature CD4 T cells, transcription factors, Runt domain

## Abstract

Runx proteins (also known as Runt-domain transcription factors) have been studied for a long time as key regulators of cellular differentiation. RUNX2 has been described as essential for osteogenesis, whereas RUNX1 and RUNX3 are known to control blood cell development during different stages of cell lineage specification. However, recent studies show evidence of complex relationships between RUNX proteins, chromatin-modifying machinery, the cytoskeleton and different transcription factors in various non-embryonic contexts, including mature T cell homeostasis, inflammation and cancer. In this review, we discuss the diversity of Runx functions in mature T helper cells, such as production of cytokines and chemokines by different CD4 T cell populations; apoptosis; and immunologic memory acquisition. We then briefly cover recent findings about the contribution of *RUNX1, RUNX2* and *RUNX3* to various immunologic diseases. Finally, we discuss areas that require further study to better understand the role that Runx proteins play in inflammation and immunity.

## Introduction

Runx is an evolutionary conserved family of transcription factors (TFs) that are best known for their roles in the regulation of the expression of genes involved in embryonic development and cell differentiation. The role of Runx proteins in hematopoietic differentiation has been broadly studied (1). Detailed reviews summarize how Runx family members orchestrate developmental processes at different stages, from stem cells to differentiation of CD4 cells into particular functional subsets (2–4). The functions of Runx proteins in embryonic development are not restricted to the blood system; these proteins are also involved in regulation of the skeletal system, nervous system, hair follicle and mammary gland development (2). Among the best characterized functions of Runx are the regulation of the cell cycle, proliferation, growth and senescence (5, 6) and the response to DNA damage and hypoxia (7, 8). The Runx family has also come to prominence due to its involvement in numerous diseases. Indeed, RUNX1/AML1 was first described as a frequent target of translocations in leukemia (9). Additionally, RUNX1 is a potential therapeutic target in various types of cancer, including myeloid and lymphoid leukemias (10, 11).

Although the Runx family has been studied mostly in the context of tumorigenesis or early hematopoiesis and thymopoiesis, recent studies suggest participation of Runx in regulating the immune response in mature T cells. Growing evidence supports the importance of Runx for long-term memory, survival and exhaustion of T cells. However, some existing data are controversial, and many unanswered questions remain in this field.

In this literature review, we summarize and conceptualize new findings of Runx functions in the T cell–mediated (CD4^+^ in particular) immune response, survival and immunologic memory acquisition and highlight new topics that warrant further study in the future.

## The RUNX Family

The mammalian Runx family includes three members: RUNX1 (AML1; CBFA2), RUNX2 (AML3; CBFA1) and RUNX3 (AML2; CBFA3). RUNX1 and RUNX3 are indispensable for normal blood cell development, and RUNX2 is a crucial regulator of bone ossification.

*RUNX1* is located on chromosome 21 (21q22.12) in humans, and *Runx1* on chromosome 16 (16qC4) in mice. Mice with a whole-body *Runx1* deletion die *in utero* due to hemorrhaging in the central nervous system and hematopoietic system failure (12, 13).

RUNX2 is a well-known master regulator of osteoblast and chondrocyte differentiation. *RUNX2* is located at 6p21.1 in humans and *Runx2* at 17qB3 in mice. Mice lacking Runx2 die immediately after birth due to complete failure of bone ossification and inability to breathe (14–17). Mutations in coding regions of *RUNX2* are associated with cleidocranial dysplasia in humans (18). In the hematopoietic system, RUNX2 has been studied mostly in plasmacytoid dendritic cells (pDCs), in which RUNX2 facilitated pDC homeostasis and anti-viral responses (19). The level of *Runx2* expression is relatively low in the T cell lineage. However, ectopic *Runx2* expression causes significant dysregulation of thymocyte maturation and increases expansion of immature CD8 cells (20).

The locations of human *RUNX3* and murine *Runx3* are at 1p36.11 and 4qD3, respectively. The majority of *Runx3* ^-/-^ mice die soon after birth with gastric epithelial hyperplasia (21). The surviving pups spontaneously develop inflammatory bowel disease with significantly increased IgA levels, disrupted TGF-β signaling in DCs and eosinophilic lung inflammation (22, 23). RUNX3 also plays a role in the development of dorsal root ganglia (24, 25). Although some studies report that RUNX1 and RUNX3 can compensate for one another, other studies show that they act in a nonredundant manner (26).

All three *Runx* genes have two alternative promoters, proximal (P2) and distal (P1), and the transcripts also undergo alternative splicing, giving rise to multiple isoforms and producing diffuse bands on Western blots. The patterns of the isoform expression are cell type–specific, time-dependent and nonredundant. For example, during the early commitment of the hematopoietic lineage, the *Runx1* short isoform generated from the proximal promoter (p*Runx1*) is predominant, whereas at the later stages of development and in adult mice, transcription of the distal promoter isoform (d*Runx1*) prevails over p*Runx1* (27). Deletion of P2 in the early mouse embryo abrogates the emergence of definitive hematopoietic progenitors, but knockout (KO) of only d*Runx1* at the same stage allows differentiation of hematopoietic precursors (28, 29).

Each of the Runx isoforms contains the highly conserved N-terminal Runt domain, which binds to the core sequence ‘YGYGGTY’ (where Y represents pyrimidine). Despite the strong homology of the Runt domain between the paralogous *Runx* genes, recent studies have described structural and functional dissimilarities that influence their DNA-binding affinities (30). The majority of the known Runx isoforms have highly related TAD (transactivation) and VWRPY domains at their C-termini (31–33).

As with other TFs, Runx proteins are subject to post-translational modifications that affect Runx cellular localization, stability, DNA-binding affinity and ability to interact with other proteins—sumoylation (34), acetylation, phosphorylation (35–37), ribosylation (38), methylation (39, 40) and ubiquitination (41). For example, methylated RUNX1 has increased transcription activation potency (39), whereas poly (ADP)-ribosylation of RUNX1 and RUNX3 enables interaction with the helicase BLM (Bloom syndrome protein) in response to DNA damage in the context of Fanconi anemia (38).

Runx proteins share the ability to dimerize with CBF beta (CBFβ) (42). Heterodimerization of Runx proteins increases their ability to bind DNA and protects them from ubiquitination (43). Additionally, a recent study demonstrated that CBFβ controls the translation of *RUNX1* (44). CBFβ does not have DNA binding activity itself but is instead recruited to DNA by all members of the Runx family through physical interactions (45). Complete loss of CBFβ is fatal and has a phenotype similar to *Runx1* ^-/-^ (46).

Among the TFs that cooperate and/or compete with the Runx proteins upon DNA binding are members of the ETS family (47), KLF4 (48), NFAT (49), AP-1 (50, 51), STAT5 (52) and many others, which may explain the multitude of Runx functions in different cell types. In this review, we do not cover all known interactions but instead highlight those most relevant to the immune system (**Table 1**). Many interaction partners are shared between all members of the Runx family, but some are specific (**Table 1**). Further in-depth exploration of RUNX1, RUNX2 and RUNX3 interaction partners is a subject for future studies.

**Table 1:** Human and murine Runx family protein domain interactions.

	RUNX1	RUNX2	RUNX3
Runt (N-terminus)	Transcription factors		
	AP-1 (50, 51)	AP-1 (50, 51)	GATA3 (53)
	ETS1 (47)	ATF4 (54)	SMADs (55)
	FOXP3 (56)	ETS (57)	STAT5 (52)
	GATA-1 (58)	LET1 (59)	TCF4 (60)
	KLF4 (48)	STAT1 (61)	
	PU.1 (62)	STAT5 (52)	
	SMADs (63, 64)	TWIST (65)	
	STAT5 (52)		
	Co-regulators		
	BRD1 and INI1 (SWI/SNF) (66)	CBFβ (33)	BRD2 (SWI/SNF) (67)
	CBFβ (45, 68)		CBFβ (45, 68)
	CDK6 (69)		JAB1/CSN5 (70)
	SUV39H1 (71)		MDM2 (72)
	MLL1 (KMT2A) (73)		PIM-1 (36)
TAD	ERK (35)	CDK4 (74)	CDK4 (5, 41)
	FOXP3 (75)	HDAC3 (76)	HDACs (77)
	HDAC1/3 (78)	HDAC4 (79)	p300 (80, 81)
	HIPK2 (82)	HDAC6 (83)	SUV39H1 (84)
	MOZ (82, 85)	MOZ (85)	
	p300 (82, 86)	p300 (87)	
	SIN3A (88, 89) (between Runt and TAD)	pRb (90)	
	SUV39H1 (84)		
VWRPY (C-terminus)	TLE/GRG (91)	TLE/GRG (91)	TLE/GRG (92)

## RUNX in Epigenetic Regulation

Due to diverse protein-protein and protein-DNA interactions, members of the Runx family can control chromatin state in many different ways in a context-dependent manner.

### Histone Methylation

Histone methylation is the chemical modification of histone protein by addition of 1-3 methyl groups to specific amino acids within the histone tail. It may recruit other proteins to chromatin, thereby altering DNA accessibility and negative or positive regulation of gene expression. H3K4, H3K9, H3K27 and H3K36 are the most studied amino acids for the modifications driven by histone methyltransferases. H3K4me1-3 and mono-methylation of H3K9 are associated with active chromatin status, whereas di- and tri-methylation of H3K9, H3K27 and H3K36 are found in abundance in silent/inactive chromatin (93).

Runx proteins can repress or activate genes through direct and/or indirect interactions with chromatin modifiers (94). In a recent study by Lee et al., RUNX3 was shown to act as a pioneer factor, cooperating with trithorax group (TrxG) and polycomb group (PcG) chromatin-modifying complexes while regulating the transition through the R (restriction)-point in HEK293 cells. The Runt domain of RUNX3 physically interacts with the bromodomain of BRD2. BRD2 acts as a bridge between RUNX3 and the MLL1/MLL5 and SWI/SNF protein complexes, which promote chromatin opening after mitotic stimulation (5). The interaction between RUNX3 and PcG occurs during later events in cell cycle; PRC2 (polycomb repressive complex 2) H3K27 methyltransferase activity is associated with decreased transcription (95). RUNX3 recruits members of PRC2 (EED and EZH2) together with histone deacetylase 4 (HDAC4), which removes permissive acethylation chromatin marks, thereby decreasing chromatin accessibility (5). These interactions are followed by recruitment by RUNX3 of CyclinD1, the key regulator of cell cycle progression, when cells pass through the R-point toward S phase (5). Although the above studies have been conducted in cancer cell lines, the molecular interactions of RUNX proteins with cell cycle machinery could be similar in all cell types.

Cooperation between RUNX1 and both the TrxG and PcG complexes occurs in early hematopoietic development: RUNX1 recruits the SWI/SNF chromatin-remodeling complex (through its BRG1 and INI1 subunits) and the MLL1 H3K4 methyltransferase to the *PU.1* gene locus and other hematopoiesis-specific gene loci (66, 96), thereby alleviating repressive methylation. Conversely, RUNX1 interaction with PRC1 causes repressive histone methylation, as demonstrated in murine thymocytes (97).

H3K9-methyltransferase SUV39H1 interaction was reported in Jurkat T cells with RUNX1 and RUNX3 but not with RUNX2 (**Table 1**). This interaction was proposed to be important for *CD4* gene silencing (84). Interestingly, SUV39H1 negatively regulates RUNX1, decreasing its DNA binding affinity when they interact *in vitro* (71, 84).

### Histone Acetylation

All Runx proteins also interact with histone acetylation complexes. Histone acetylation is generally associated with active chromatin and is catalyzed by enzymes called histone acetyltransferases (HATs). bRUNX1 isoform and its partner CBFβ interact with HAT p300 and, together with the tumor suppressor PML, homeodomain-interacting protein kinase 2 (HIPK2) and monocytic leukemic zinc finger (MOZ) HAT, form a chromatin regulatory complex that increases the expression of target loci in murine hematopoietic cells progenitors (82). RUNX1 may play a role not only as a sequence-specific activation factor, but also as a scaffold in this multiprotein machinery (98). Choi et al. showed that *Runx1* deletion reduces permissive H3K27ac and decreases chromatin accessibility in mouse leukemia cells (T-ALL) (10). Phosphorylation of RUNX1 is required for the efficient assembly of the complex with histone acetyltransferases (82).

### Histone Deacetylation

While acetylation on histones (such as H3K9ac, H3K27Ac) correlates with increased DNA accessibility, loss of this chromatin mark results in inhibition of gene expression. Both Runt and TAD domains interact physically with histone deacetylases (HDACs) (**Table 1**). HDAC1 and HDAC3 bind RUNX1 directly through the TAD domain and, together with suppressive histone methyltransferases, such as SUV39H1, negatively regulate Runx targets (78, 84).

In addition to HDACs Runx can also recruit transcription repression complexes such as Gro(Groucho)/TLE. This interaction was proposed as essential for *Cd4* silencing in mouse thymocytes and splenocytes by Yarmus et al. (92). Another study (91) demonstrated that disruption of interactions between RUNX1, RUNX3 and Gro/TLE resulted in partial depression of *Cd4* and *ThPOK* genes in developing CD8 T cells.

Together these findings clearly point to a critical role for direct and indirect cooperation between Runx family members and histone modifiers, though further studies are needed on the understudied mechanisms of chromatin accessibility regulation by the members of Runx family.

### DNA Demethylation

One of the well-studied epigenetic mechanisms of gene expression is DNA methylation. In mammals, methyl groups are typically found in CpG-rich regions and cause repression of gene transcription when located in promoters. The opposite process—removing methyl marks from DNA—may happen passively during cell division or actively through an enzymatic reaction. DNA demethylation is a highly important process for chromatin opening in development and for forming the epigenetic landscape of immunological memory (99, 100). RUNX1-mediated epigenetic regulation involves not only histone-modifying complexes, but also recruitment of DNA-demethylating machinery. Co-immunoprecipitation experiments have revealed interactions between RUNX1 and proteins from the family of ten-eleven translocation methylcytosine dioxygenases (TET2 and TET3) in the Jurkat T-lymphocyte line (101). Further, active demethylation of RUNX1 DNA-binding sites, including in the RUNX1 promoter itself, has been observed in HEK−293T cells overexpressing RUNX1 (101, 102). RUNX1 motif overrepresentation in demethylated regions was also shown for human peripheral blood CD34^+^ hematopoietic progenitor cells and CD14^+^ monocytes.

Additional studies on primary cells are needed to delineate the mechanisms of RUNX-mediated DNA methylation and demethylation control in the different stages of hematopoietic lineage development and normal homeostasis of fully differentiated lymphocytes and to elucidate the association of these processes with diseases.

### Other Nuclear Functions

Runx TFs also have other nuclear functions that are not related to histones or DNA modification. For example, a significant portion of RUNX3 in a dividing cell binds to mitotic structures when the Runt domain is phosphorylated by the cell cycle regulatory Aurora kinases, resulting in a loss of DNA-binding ability. This RUNX3–mitotic structure interaction is crucial for on-time mitotic progression, as it was shown in several cancer cell lines (103). Another remarkable function of Runx related to cell division is mitotic memory, which was first demonstrated in a human osteosarcoma cell line. The RUNX2 protein associates with chromatin with sequence specificity (through the conserved Runt domain) during mitosis, helping to transmit epigenetic memory to progeny cells (104, 105). A similar finding has also been described for RUNX3 in dividing gastric epithelium cells (106). In interphase, the subnuclear localization of Runx proteins is defined by the C-terminal signal (106, 107). Interaction with nuclear matrix is critical for Runx function in early development: deletion of the nuclear matrix targeting signal in RUNX2 resulted in a failure of osteoblast maturation and dramatically impaired skeleton development (108). Dissociation from the nuclear matrix may be triggered by ERK-induced phosphorylation of residues located between the Runt and TAD domains, as it was shown for RUNX1 (88).

Finally, Runx proteins appear to be involved in spatial chromatin organization. RUNX1 cooperates with STAG2—a subunit of the cohesion complex—to establish a distal promoter-enhancer interaction in hematopoiesis (109). The connection of distal DNA regulatory elements to promoters by RUNX1 has also been demonstrated for the *CD34* gene in hematopoietic stem cells and for *Tcrb* and the TCR β enhancer (Eβ) in thymocytes (110, 111).

Taking all the pieces together, we can see that the spectrum of mechanisms by which Runx carries out its regulatory function on an epigenetic level is broader than was thought before: from cooperation with histone-modifying complexes and DNA-demethylating enzymes to participation in 3D chromatin folding. Nevertheless, many new interactions may yet remain undiscovered. It is still unknown whether this nuclear function are similar for Runx proteins T cells, noticing the existence of different Runx isoforms and post-translational modifications, but studies in this field may reveal more details about how Runx regulates mature T cells function.

## RUNX in the Cytoplasm

The Runx proteins predominantly localize to the nucleus, but a small fraction can be detected in the cytoplasm. For example, an inhibitory role of cytoplasmic RUNX1 on the NF-κB pathway has been reported by Nakagawa et al. NF-κB signaling is one of the central pathways regulating the inflammatory response and immune cell differentiation (112–114). In the cytoplasm, RUNX1 physically binds to the central regulator of NF-κB activation, IKKß, attenuating downstream signaling and proliferation of hematopoietic cells (114). A similar mechanism for preventing an excessive inflammatory response to bacterial infection has also been described in respiratory epithelial cells (115). In contrast, in macrophages, RUNX1 co-immunoprecipitates with p50—the subunit of the canonical NF-κB heterodimeric complex—and acts as a positive regulator of inflammatory TLR4-mediated NF-κB signaling (116). These contradictory results demonstrate how the role of RUNX1 varies depending on the cell type. Further studies are needed to investigate the relationship between the cytoplasmic fraction of Runx proteins and NF-κB in the inflammatory response of T cells.

Due to the absence of a nuclear-export signal, Runx proteins are likely transported to the cytoplasm through other mechanisms. Jun activation domain–binding protein 1 (JAB1/CSN5) has been identified as a mediator of the nuclear export of RUNX3 to the cytoplasm, where RUNX3 is rapidly degraded by proteasomes (70). Kim et al. proposed that RUNX3 translocation correlates with phosphorylation of its serine and threonine (Ser/Thr) residues within the Runt domain by the proto-oncogene Ser/Thr kinase Pim-1 and may be associated with cancer (36). The phosphorylation stabilizes RUNX3, sequesters it from the nucleus and disrupts its transactivation functions (36). Another study in multiple tumor cell lines (including HeLa, SYF, HEK-293T, MKN45, SNU16, BT20 and MB468) showed that RUNX3 may be phosphorylated by Src kinase, resulting in mislocalization; Src was predominantly localized in the cytoplasm but also found in nucleus, and when Src was inhibited by siRNAs, RUNX3 was able to translocate back to nucleus (37). Src-mediated nuclear export may be triggered by oxidative stress (117). Finally, Runx proteins can potentially shuttle between the nucleus and cytoplasm by N-terminal–mediated association with microtubules, as was demonstrated for RUNX2 in HeLa cells (118). Cytoplasmic translocation interferes with Runx tumor-suppressing functions and has been found in patients with gastric cancer (119) and breast cancer (120), emphasizing the likely importance of Runx localization in human disease processes.

## RUNX in the Development of T Lymphocytes

The Runx proteins play distinct roles in promoting thymocyte differentiation during several stages, as summarized in recent reviews (2, 121). RUNX1 controls the transition of immature T cells from the double-negative (DN) to double-positive (DP) population stage by regulating expression of the *Tcrb* gene (111) among others. Subsequently, RUNX1 and RUNX3 are involved in CD4^+^ or CD8^+^ single-positive cell commitment through repression of Thpok and gene silencing of *Cd4* at the DP stage (122, 123). In mature CD8 cells, RUNX1 and RUNX3 synergistically cooperate to suppress *Cd4* gene expression (91). Inhibition of RUNX1 at the DP stage leads to skewing of the population to CD8 T cells, whereas RUNX3 deficiency results in a decreased number and compromised function of CD8 cytotoxic T lymphocytes (CTLs) (13, 124, 125). RUNX3 binds to *Cd8* enhancer *E8I* activating and maintaining *Cd8a* gene expression in mouse effector CD8 T cells (126). In addition to its well-described role in CD8 T cells, RUNX3 also participates in CD4 T helper subsets differentiation (53, 127, 128). *Runx2* expression is mostly restricted to the earliest DN population in embryonic development. Enhanced *Runx2* expression during the β-selection stage in the developing thymus resulted in increase of immature single-positive CD8 T cells (20).

## RUNX and Effector Functions of CD4 T Cells

**Figure 1:**
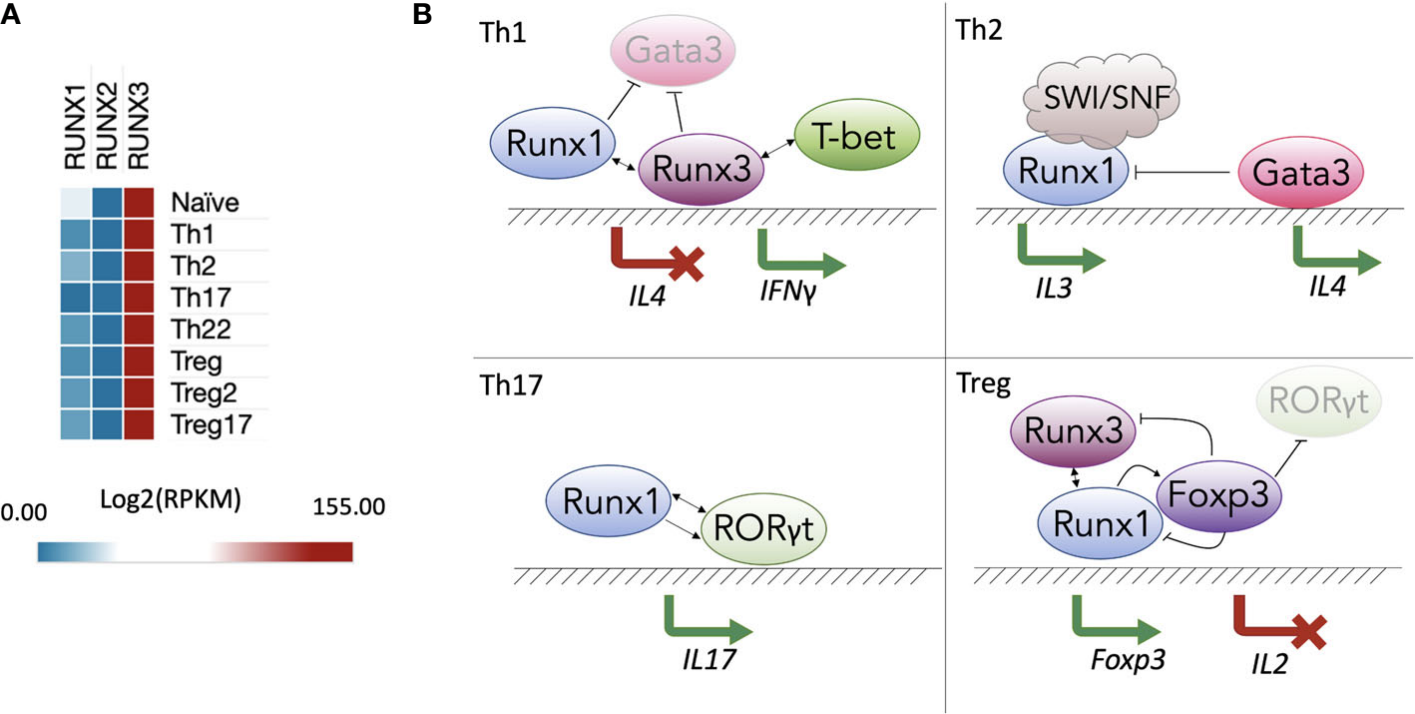
*RUNX* gene expression and function in T helper populations. **(A)** Expression of Runx proteins in different Th subsets of human CD4^+^ T cells (129) (GSE149090) processed in SciDAP (https://scidap.com, Datirium); **(B)** Runx-mediated Th differentiation. In the Th1 subtype (top left), RUNX1 and RUNX3 compete with GATA3 and suppress expression of Th2-signature cytokines, such as IL-4. RUNX3 and T-bet induce *IFNG* expression. In the Th2 subtype (top right), RUNX1 interacts with the SWI/SNF complex to induce *IL3*. In the Th17 subtype (bottom left), RUNX1 and RORγt cooperate to induce expression of *IL17*. In T regulatory cell (Treg) populations (bottom right), RUNX1 is essential for transcription of *FOXP3*, whose protein product forms a complex with RUNX1 and RUNX3 to inhibit *IL2*.

Upon TCR activation, naïve CD4 T cells differentiate into various T helper (Th) subsets. Th type-specific commitment depends on the cytokine milieu, and each Th subset has a specific cytokine expression pattern. Although the Runx-CBFβ complex is broadly involved in regulating cytokines in different Th subtypes, the transcription of each Runx gene in resting human CD4 T cells does not vary much between Th subsets, with RUNX3 being the most highly expressed on the mRNA level (129) (**Figure 1A**). In the following section, we describe the known roles of Runx proteins in the different lineages of CD4 T cells and in effector CD8 T cells.

### Th1 Subset

The Th1 lineage is of key importance for the immune response to intracellular viral and bacterial infections. The T cells of this subset differentiate under control of the transcription master regulator T-bet and secrete IL-2, IFNγ and TNF-α cytokines when they encounter pathogens (130). In a *Runx1* KO, CD4 cells display a severe block in T cell maturation and compromised response to TCR activation. For instance, *Tnfa* expression in response to α-CD3ϵ/α-CD28 stimulation is substantially reduced by *Runx1* conditional KO in peripheral CD4 cells (131). Future studies are needed to fully elucidate the underlying mechanisms of how much the absence of Runx can affect TNF-α production in fully differentiated Th1 cells.

During Th1 polarization, the genes of non-Th1 cytokines, such as IL-4, are silenced. The Runx proteins bind regulatory elements and inhibit *IL4* gene expression, which makes them suppressors of the Th2 lineage. A “functional compensation” mechanism between RUNX1 and RUNX3 has been proposed, wherein RUNX1 is bound to the *Il4* silencer in naïve CD4 T cells; during primary activation and differentiation, RUNX3 replaces RUNX1 and promotes skewing to the Th1 population (132). RUNX3 not only inhibits Th2 lineage differentiation but also increases expression of Th1-specific effector molecules, such as IFNγ. IFNγ is a critical cytokine mostly produced by Th1, cytotoxic T cells and innate immune cells, such as natural killers (NKs), that activates of Jak-STAT pathway in macrophages, NKs and neutrophils (133). dRUNX3 (an isoform produced by the distal *Runx3* promoter) induces transcription of *Ifng* by binding to its promoter together with T-bet (128, 134, 135). As a result, RUNX3 and T-bet promote polarization of naïve T cells to the Th1 subtype.

In inflammatory conditions, Th1 cells can be converted into Th17 cells by TGF-β/IL-6 treatment. In mice, TGF-β/IL-6 treatment causes the opening of chromatin around RUNX1 and RORγt binding sites in the promoters of the *Il17* and *Rorc* genes, resulting in IL-17 expression. Knockdown of *Runx1* prevents the trans-differentiation of the Th1 cells into the Th17 phenotype (136). Finally, in Th1-like Th17 cells, which produce both IL-17 and IFNγ, RUNX1 initiates transcription of *Ifng* together with the lineage master regulator T-bet in a similar manner to that of RUNX3 in classic Th1 cells (**Figure 1B**, top left panel) (134, 137). In studies examining CD4 T cell quiescence, KO of *Runx1* in mouse total resting CD4 T cells does not produce changes in *Ifng* expression (138, 139), whereas downregulation of *Runx3* in Th1 cells leads to decreased *Ifng* production (53). Thus, there may be cooperative behavior between RUNX1 and RUNX3 in the regulation of *Ifng* expression.

### Th2 Subset

RUNX1 plays an important inhibitory role in the differentiation of naïve CD4 T cells towards the Th2 lineage, with RUNX1 overexpression repressing the production of the Th2-specific cytokines IL-4 and IL-5 (140). Elevated levels of secreted IL-4 and IL-5 and decreased levels of IFNγ and IL-2 during the early phase of TCR activation occurs in mouse CD4 T cells expressing the transgenic Runt domain, which is a dominant-negative form of Runx (RunxDN) (140). Likewise, in Th1 cells RUNX1 suppresses GATA3, which plays a central role in initiating Th2 lineage commitment (140). In contrast, during Th2 differentiation, GATA3 replaces the Runx-CBFβ complex on the *Il4* silencing element (132), leading to cytokine induction (**Figure 1B**, top right panel). Th2 cytokines (IL-4 and IL-13) and *Gata3* are also upregulated in peanut allergen–challenged *Runx3* ^+/-^ murine polarized T cells compared to peanut allergen–challenged wild-type murine polarized T cells (141). Interestingly, in the group 2 innate lymphoid cells (also known as Th2-like ILC2s), deletion of RUNX1 and RUNX3 causes the opposite effect on IL-13—it compromises the production of the Th2 cytokines IL-5 and IL-13 and enhances the production of IL-10, inducing an exhaustion-like phenotype (142). It indicates that RUNX1 and RUNX3 may influence expression of the same effector molecules but in a different way in other lineages. To summarize the recent research in T helper CD4 cells, RUNX1 and RUNX3 cooperate with (or compete for DNA binding with) lineage master regulators, and sometimes cooperate with each other to negatively regulate Th2 polarization.

### Th17 and Tregs

The pathways involved in the development of Th17 and Treg cell subsets are intertwined. The master regulator of Th17 differentiation, RORγt, competes with FOXP3—the key TF in Treg lineage development—for binding to regulatory elements in differentiating CD4 T cells. RORγt and RUNX1 cooperatively bind the *Il17* promoter and its enhancer CNS-5 in *in vitro*–polarized Th17 cells (**Figure 1B**, bottom left panel) (75). RunxDN strongly suppresses expression of IL-17 (75).

FOXP3, a suppressor of IL-17 and antagonist of RORγt, directly binds the RUNX1 protein, repressing the inflammatory phenotype of Th17 cells (75). However, it is not completely clear whether they bind DNA simultaneously and operate as a single complex. Meanwhile, in Treg cells, the Runx-CBFβ complex acts as an essential transcriptional activator of *Foxp3* (56, 127, 143). *Cbfb* ^fl/fl^ Treg cells show significantly reduced anti-inflammatory function (56, 127, 143, 144). Interestingly, small amino acid sequence differences in the Runt domains result in RUNX3 having higher binding affinity to the *Foxp3* promoter than RUNX1 (30).

Physical interactions between RUNX1 and FOXP3 are also required for efficient *Il2* inhibition (**Figure 1B**, bottom right panel). In a model of Treg cell differentiation proposed by Ono, FOXP3 reduces RUNX1-dependent transcriptional activation of *Il2*. Without FOXP3, RUNX1 binds to *Il2* and many other promoters and enhancers of genes required for the TCR-mediated response, making them primed for secondary AP-1/NFAT/NF-κB–inducible expression (121, 144). RUNX1 and RUNX3 act in a cross-compensatory manner to enhance and maintain Treg cell function. Nevertheless, *in vivo* studies illustrate that inhibiting RUNX1, but not RUNX3, induces lymphoproliferation, severe autoimmune reactions and hyperproduction of IgE (145). This phenotype is similar to the phenotype observed in *Foxp3*-deficient mice (145–147).

Severe lung inflammation occurs in mice with a *Runx1*-deleted *Bcl2*-transgenic (which was introduced to improve CD4 cell survival) naïve CD4 T cells. Mice experience lymphocyte infiltration in the lungs with consequent spread to other organ systems. Interestingly, *Runx1* ^-/-^ cells in this model display spontaneous activation and elevated expression of IL-17 and IL-21, despite the fact that RUNX1 is usually considered to be a positive regulator of IL-17 during Th17 development (138). The authors discussed that enhanced level of IL-21 might increase IL-17 expression indirectly influencing the inflammation response (138, 148).

### Tfh

The study by Choi et al. demonstrated that Runx proteins are involved in the process of differentiation of Tfh (T follicular helper) cells – a specialized subset of CD4 T cells responsible for providing instructing signals to B cells in germinal centers (149). The study identified that RUNX2 and RUNX3, together with GATA3, and Klf2, are under negative control of TF Bcl-6 and regulate expression of genes important for proper Tfh function including production of IL-21 and IL-4 (149). This “repressor-of-repressors” Bcl-6 gene regulatory network displays the complexity of transcriptional control of Tfh differentiation where Runx proteins play critical role.

### Th22

Th22 cells produce IL-22, which exerts pro-inflammatory influences on epithelial cells (150, 151). A recent study by Sekimata et al. showed that RUNX1 and RORγt bind to the *Il22* enhancer and synergistically enhance its expression upon naïve T cell activation (151).

### Other Cytokines

Several studies present *Runx1* KO data in which *Il2* mRNA levels are significantly upregulated, whereas others report RUNX1 as a positive regulator of IL-2 production. In the model suggested by Wong et al., *Il2* is repressed in murine resting naïve T cells by the RUNX1 distal isoform (dRUNX1). During early TCR stimulation, calcineurin-NFAT signaling induces expression of another RUNX1 isoform, the shorter proximal RUNX1 (pRUNX1). pRUNX1 acts in an autoinhibitory manner and represses dRUNX1. When dRUNX1 is removed from the *Il2* promoter, the inflammatory signaling is enabled (152, 153). In contrast, Ono et al. demonstrated in Jurkat and mouse total CD4 T cells that RUNX1 binds to the *Il2* promoter and activates its expression. Knockdown of RUNX1 led to significantly reduced IL-2 production in activated cells (144). Murine CD4 T cells depleted of RUNX3 also showed a substantial decrease in IL-2 expression (134). The contradiction between these results may be explained by the use of different models, approaches and time points when IL-2 expression was examined. More studies are needed to better understand the relationship between RUNX1, RUNX3 and IL-2 in different contexts.

IL-3 is another highly TCR-inducible cytokine and is preferentially expressed by Th2 cells (154). Multiple studies have shown that RUNX1 binds to the *Il3* region and is able to transactivate the *Il3* promoter (155, 156). For example, in the Jurkat cell line, RUNX1 recruits the SWI/SNF chromatin remodeling complex to establish robust *IL3* transcription (**Figure 1B**, top right panel) (66).

### Chemokines

The CC chemokines are the major attractants that induce immune cell migration to areas of inflammation. For example, CCL5 attracts T effector and T resident memory cells. The CCL3, CCL4 and CCL5 chemokines are upregulated in CBFβ-depleted, activated T cells (157). Further, CCL5 is expressed at higher levels in *Runx3* ^-/-^ compared to *Runx1* ^-/-^ CD4 T cells. The Runx-CBFβ complex binds to the *Ccl5* enhancer and, together with the chromatin organizer SATB1, restrains the expression of *Ccl5* (157). Positive regulation of CCL3 and CCL20 by RUNX3 contributes to the recruitment of CD8 T cells into the microenvironment of lung adenocarcinoma, supporting anti-tumor immunity (158).

## RUNX and Effector Functions of CD8 T Cells

Mature cytotoxic CD8 T cells lacking RUNX3 not only showed depression of *Cd4*, but possess substantially impaired proliferation in response to CD3/CD28 activation (124). RUNX3 is also necessary for effector molecules production in CTLs: isolated mouse *Runx3^-/-^* CD8 T cells in culture produced significantly lower amount of IFNγ, perforin and granzyme B than wild type cells in response to activation. It was shown that RUNX3 directly binds regulatory elements of *Ifng*, *Gzmb* and *Prf1* and therefore required for effective cytolytic function of mature CD8 T cells (159). In absence of RUNX3 transcriptional program of cytotoxic CD8 T cells moves towards Tfh phenotype because of de-repression of Tcf7 and aberrantly high expression of Bcl-6 impairing its normal function and signaling (160).

To summarize, RUNX1 and RUNX3 are essential regulators of Th lineage differentiation and have T cell subset–specific functions. Together with lineage master regulator TFs, Runx proteins are intertwined in a complex network of transcription and epigenetic regulation of cytokines and chemokines in differentiating and mature Th cells and cytotoxic CD8 T cells.

## RUNX and Immunologic Memory

Immunologic memory is the ability of the immune system to “remember” previous encounters with pathogens and develop a rapid response to a secondary challenge. Long-lived memory T cells are one of the most important players in the adaptive immune response—they are able to undergo massive proliferation, giving rise to numerous effector cells. There is growing evidence of the importance of Runx proteins in the differentiation of T cells into effector and memory populations.

We have previously proposed that immunologic memory is encoded epigenetically (161) *via* the presence of areas of open chromatin in the vicinity of rapid recall genes in memory, but not naïve T cells. Runx-binding sites are frequently found within accessible chromatin regions of both memory CD4 and CD8 T cells (together with ETS family, Sp1 and T-bet motifs) (155, 162). Upon activation, inducible TFs, such as AP-1, NFAT1 and NF-κB, bind to silent chromatin in naïve T cells and initiate cytokine signaling. The hypothetical mechanism of long-term immune memory is that constitutive TFs, such as RUNX1 and ETS1, are bound to the opened regions and maintain the chromatin in an accessible state, poised for the next encounter (155, 163, 164).

Several prominent studies were conducted to show the role of Runx proteins in CD8 T memory cells. Wang et al. showed that RUNX3-induced changes in chromatin accessibility were required for differentiation of CD8 CTLs into an effector memory cell subset (165). Another study (166) determined that RUNX3 regulates the transcriptional program of CD8 T memory cells’ tissue residency. Gene expression profiling of the CD8 T cell population revealed that RUNX3 promoted expression of tissue-residency signature genes and attenuated the ability of cells to circulate in blood flow (166). Another member of the family, RUNX2, was also found to be important for memory T cells. Olesin et al. demonstrated that *Runx2* KO results in a significant loss of LCMV-specific CD8 memory cells without impairing the rapid recall response in mouse (167).

While the studies indicate an essential role of RUNX2 and RUNX3 functions in CD8 T memory cells, expression of *Runx3* may be interfering with long-term memory in CD4 cells. Ciucci et al. proposed that maintaining anti-viral memory potential in a population of long-lived CD4 T cells requires downregulation of *Runx3* and *Prdm1* by ThPOK (168). In particular, RUNX3 and BLIMP promote the short-lived effector cell subset that is derived from T central memory progenitor (Tcmp) cells in response to viral infection in mouse. However, in Tcmp cells, ThPOK did not allow the initiation of the suppressive, exhaustion-like effector transcriptional program, which may be driven by RUNX3 and BLIMP (168).

Thus, all three members of the Runx family participate in processes related to immunologic memory in T cells but from the different perspectives of promoting effector or long-lived central memory phenotypes and cell survival. However, many questions remain unanswered, for instance whether Runx proteins are responsible for maintaining long-lived memory in various CD4 T subsets and how and when RUNX2 and RUNX3 communicate in CD8 cells.

## RUNX in Proliferation and Apoptosis of T Cells

A substantial decrease in lymphoid cell number is common in mouse models with conditionally deleted *Runx1* in T cells (125, 138, 169). This phenotype may be explained by enhanced apoptosis of CD4 T cells. However, introduction of transgenic *Bcl2* increases CD4 T cells number and survival in a *Runx1*-KO model, implicating RUNX1 in programmed cell death signaling (138). The proliferation potential is also dampened in *Runx1*-negative CD4 T cells (170), as well as in cells with an introduced RunxDN form (13). In both systems, T cells were also more susceptible to TCR activation–induced cell death than were wild-type cells (125).

On the other hand, RUNX1-deficient hematopoietic stem cells show slow growth, significantly inhibited ribosome biogenesis and reduced apoptotic activity (171, 172). Therefore, deletion of RUNX1 in hematopoietic progenitors and in mature T cells may cause different effects on the programmed cell death pathway. Subsequently, Runx proteins have a dual role in apoptosis and tumorigenesis, acting both as tumor suppressors or oncogenes in various contexts (173).

p53 has been referred to as the “cellular gatekeeper” because of its ability to initiate programmed cell death. In normal homeostasis, p53 represses transcription of dRUNX1 in T cells (170). Under stress conditions, p53 disassociates from the distal *Runx1* promoter, enabling expression of the dRUNX1 isoform, whose upregulation promotes proliferation of T cells (170). Similarly p53 deletion leads to an increase in RUNX1 levels which, in turn, contributes to the development of T cell malignancy (170). A similar relationship between RUNX3 and p53 has been proposed in a model in which loss of p53 causes RUNX3 overexpression and enhanced MYC signaling (174). RUNX3 and p53 form a negative feedback loop: RUNX3 upregulates p53 in response to stress until p53 inhibits RUNX3 (174). Importantly, ubiquitin ligase MDM2, a major antagonist of p53, also directly interacts with RUNX3. MDM2 ubiquitinates the Runt domain, directing RUNX3 to proteasomal degradation (72).

To conclude, RUNX1 and RUNX3 are involved in a very complex and delicate regulation of the cell cycle, a disruption of which can lead to T cell malignancy.

## RUNX in Diseases

Runx proteins are coming to prominence in immunology research because of their multiple roles in inflammatory disorders. In this section, we briefly review various conditions associated with RUNX1, RUNX2 and RUNX3 dysregulation.

### RUNX1

*RUNX1* is also known as *AML1* (Acute Myeloid Leukemia gene 1) because mutations, in most cases translocations, in this gene are frequently observed in patients with lymphoblastic and myeloid leukemia (9, 172). Mutations of RUNX1 often become a first-hit mutation in pre-leukemia conditions or myeloid dysplastic syndrome. Fusion proteins, such as AML1-ETO (RUNX1-RUNX1T1), are the frequent results of chromosomal translocations in leukemia. This aberrant product, as well as several other known RUNX fusion proteins, cause major pathway dysregulation and development of cancer (175–178). AML1-ETO induces profound proliferation of undifferentiated pre-leukemic cells with compromised DNA-repair machinery (179).

RUNX1 deficiency is associated with familial platelet disorder with predisposition to myeloid leukemia (FPDMM) coupled with thrombocytopenia and significant reduction in B-lymphoid, T-lymphoid and myeloid lineages (180–182). Different congenital mutations—from point substitutions to large deletions—are associated with various platelet abnormalities, including defective δ-granule release, thrombocytopenia and inherited bleeding, which determine the severity of FPDMM (180, 183, 184).

CD4 T cells with deleted *Runx1* are prone to spontaneous activation, resulting in the development of a fatal autoimmune lung disease in mice. An increased production of IL-17 and IL-21 is observed in the hyperactivated cells, which preferentially localize to the lungs. When aged, these mice experience systemic inflammation and increased expression of proinflammatory cytokines (138). Asthma-like symptoms have also been described in the *Cbfb^-^* ^/-^CD4^Cre^ animal model (132).

*RUNX1* single-nucleotide polymorphisms (SNPs) are associated with immunologic and autoimmune diseases, such as rheumatoid arthritis (185), psoriasis (186) and asthma (187, 188). RUNX1 deficiency inevitably affects its downstream targets. We analyzed the overlap of RUNX1 chromatin immunoprecipitation sequencing (ChIP-seq) peaks in Jurkat T cells (189) with SNPs in the GWAS catalog (190) using the regulatory element locus intersection (RELI) tool (191) (**Table 2**). This approach identified several significant overlaps with immunologic disease–related mutations, supporting RUNX1 as a potential therapeutic target for a spectrum of inflammatory conditions.

**Table 2:** Regulatory element locus intersection (RELI) analysis (191) of the overlap between RUNX1 Jurkat T cell chromatin immunoprecipitation sequencing (ChIP-seq) peaks and disease-risk single-nucleotide polymorphisms (SNPs) in the blood and immune system.

Phenotype	% overlap (observed n/total n)	Adjusted p-value
Mixed phenotype: chronic inflammatory diseases, ankylosing spondylitis, Crohn disease, psoriasis, primary sclerosing cholangitis, ulcerative colitis, pleiotropy	21% (45/215)	1.59E-12
Crohn disease	24% (39/167)	4.59E-08
Systemic lupus erythematosus	25% (24/96)	6.28E-08
Celiac disease	33% (14/43)	2.67E-07
Inflammatory bowel disease	20% (40/197)	7.63E-07
Multiple sclerosis	22% (27/121)	2.06E-06
Asthma	18% (22/121)	5.78E-06
Allergic disease, asthma hay fever or eczema	17% (23/136)	1.8E-04
Rheumatoid arthritis	14% (17/122)	1.32E-03
Chronic lymphocytic leukemia	17% (8/48)	2.72E-03

The top 10 most significant genome-wide association study (GWAS) terms are shown. ChIP-seq data from Jurkat cells were obtained from the GEO database, accession number: GSM1697879 (189) and processed using the BioWardrobe software package (192, 193).

Finally, RUNX1b (the distal isoform of RUNX1), is known to interact with the accessory protein 3b of SARS-CoV, which might be an interesting subject for future investigations into the Runx family’s contribution to the viral infection response (194).

### RUNX2

Because RUNX2 is extremely important in osteogenesis, deficiency of *Runx2* expression leads to bone formation defects (14, 195–197). Increased RUNX2 is associated with various malignancies, including myeloma, B-cell lymphoma, acute lymphoblastic leukemia and several others (198–201). Upregulation of RUNX2 in vascular cells may lead to calcification and increased stiffness of the vessels (202–204). A recent, comprehensive review by Chen et al. described the role of Runx2 in atherosclerosis (205). Polymorphisms in the *RUNX2* gene are associated with cleidocranial dysplasia (18, 197, 206) and osteoarthritis (207). Not much is known about the contribution of RUNX2 to immune disease. However, a recent report discussed its possible participation in asthma development, showing drastically high RUNX2 expression in lung epithelium cells (208).

### RUNX3

A recent review (209) discussed the relationship between RUNX3 and asthma: reduction of RUNX3 function *via RUNX3* hypermethylation (210) or mislocalization of the protein (in the mouse model) (211) was found to be connected with the pathogenesis of asthma. This association may be explained by the essential role of RUNX3 in the Th1/Th2 differentiation control and its induction of *Foxp3* expression in Treg cells (146). When the balance is substantially shifted toward a Th2 response, Treg cells with a compromised FOXP3 level cannot suppress the excessive secretion of Th2 cytokines, and allergic reactions may occur (209).

Hypermethylation of the *RUNX3* promoter, with most of the published data showing significance of distal promoter methylation, is a frequent finding for several cancers (120, 212–215). In contrast, hypomethylated *RUNX3* is found in patients with systemic lupus erythematosus (216). Repressed transactivation and impaired anti-tumor functions may be also due to RUNX3 mislocalization, which is frequently observed in gastric and breast cancer (119, 120). Finally, deletions in *RUNX3* gene can also result in malignancies of T cells (217). Among the phenotypes associated with polymorphisms in the *RUNX3* gene locus are ankylosing spondylitis (218–220), psoriatic arthritis (221), Crohn disease (222), asthma (223) and multiple sclerosis (224). In B cells, RUNX3 is upregulated by the Epstein-Barr virus–encoded TF EBNA2, leading to accelerated proliferation of infected cells (225).

To summarize, Runx TFs are involved in many processes in immune cells on different levels, including epigenetic, and disturbing these complex networks may impair the cell cycle, differentiation and effector molecule production, which can result in an inadequate inflammatory response or malignancy.

## Conclusion

The Runx proteins have come to prominence because of their tremendous impact on hematopoiesis and development of various cancer types. An increasing number of studies also highlight the significance of RUNX1 and RUNX3 in T cell homeostasis and the adaptive immune response. However, because Runx proteins act in a context-dependent manner, there are many contradictory results and hypotheses related to the mechanisms underlying the role of Runx in mature T cell function, differentiation into effector or memory subsets and switching between different T helper subtypes. The specific roles of RUNX1 versus RUNX3 in these processes need to be defined in human CD4 T cells: does RUNX1 act synergistically with RUNX3? In which cases do they compensate for each other and in which cases are they indispensable?

Despite major advances in biomedicine in recent years, serious epidemics, such as COVID-19 and Ebola, continue to emerge all over the world. It is clear that further research in the field of immunologic memory will be critical for developing new, effective drugs and vaccines and treatment strategies. Therapeutic regulation of specific T cell memory is also a promising possibility for cancer immunotherapy. In light of the importance of the Runx family for the rapid secondary response, the knowledge summarized herein, together with further studies, will likely be valuable for improving vaccination and immune-modulatory anti-cancer therapy.

## Author Contributions

SK and AB conceived the idea and wrote the review. SP and MW collaborated on RELI analysis. All authors contributed to the article and approved the submitted version.

## Funding

This work was supported by NIAID, NIH grant R01 AI153442.

## Conflict of Interest

AB is co-founder of Datirium, LLC. Datirium provides installation and support services for the BioWardrobe and SciDAP platforms used in this paper.

The remaining authors declare that the research was conducted in the absence of any commercial or financial relationships that could be construed as a potential conflict of interest.

## Publisher’s Note

All claims expressed in this article are solely those of the authors and do not necessarily represent those of their affiliated organizations, or those of the publisher, the editors and the reviewers. Any product that may be evaluated in this article, or claim that may be made by its manufacturer, is not guaranteed or endorsed by the publisher.
